# Combined Versus Detailed Evaluation Components in Medical Student Global Rating Indexes

**DOI:** 10.5811/westjem.2015.9.27257

**Published:** 2015-11-12

**Authors:** Kim L. Askew, James C. O’Neill, Brian Hiestand, David E. Manthey

**Affiliations:** Wake Forest School of Medicine, Department of Emergency Medicine, Winston-Salem, North Carolina

## Abstract

**Introduction:**

To determine if there is any correlation between any of the 10 individual components of a global rating index on an emergency medicine (EM) student clerkship evaluation form. If there is correlation, to determine if a weighted average of highly correlated components loses predictive value for the final clerkship grade.

**Methods:**

This study reviewed medical student evaluations collected over two years of a required fourth-year rotation in EM. Evaluation cards, comprised of a detailed 10-part evaluation, were completed after each shift. We used a correlation matrix between evaluation category average scores, using Spearman’s rho, to determine if there was any correlation of the grades between any of the 10 items on the evaluation form.

**Results:**

A total of 233 students completed the rotation over the two-year period of the study. There were strong correlations (>0.80) between assessment components of medical knowledge, history taking, physical exam, and differential diagnosis. There were also strong correlations between assessment components of team rapport, patient rapport, and motivation. When these highly correlated were combined to produce a four-component model, linear regression demonstrated similar predictive power in terms of final clerkship grade (R^2^=0.71, CI_95_=0.65–0.77 and R^2^=0.69, CI_95_=0.63–0.76 for the full and reduced models respectively).

**Conclusion:**

This study revealed that several components of the evaluation card had a high degree of correlation. Combining the correlated items, a reduced model containing four items (clinical skills, interpersonal skills, procedural skills, and documentation) was as predictive of the student’s clinical grade as the full 10-item evaluation. Clerkship directors should be aware of the performance of their individual global rating scales when assessing medical student performance, especially if attempting to measure greater than four components.

## INTRODUCTION

Most medical schools employ a combination of multiple choice testing, standardized patients, and direct observation to evaluate their students’ performance in terms of knowledge, skills, and attitudes.^1^ A staple among evaluations of medical students in clinical rotations is the assessment of a student by a faculty member or resident using a global rating scale (GRS) with varied components, such as knowledge, rapport, procedural skill and documentation quality. Distinct from other clerkships, most EM clerkships require the use of a GRS evaluation card on every shift because students interact with multiple faculty members over the course of a rotation, as opposed to a sustained interaction with one or two individual faculty members.^1^ Most clerkships use these cards because students work with different faculty each shift and because of evidence that shift cards promote immediate and satisfactory feedback.^2^,^3^ Since students do not consistently work with the same attending and residents over a two- to four-week period, clerkship directors rely on the formative feedback provided on shift cards to gauge a student’s aggregate clinical performance.

Over the past six decades, the use of these subjective evaluations has found critics and advocates.^4^ In order to maximize feedback and quality of evaluations, many institutions have gone to a criterion-based multi-point scale for multiple attributes. However, problems with multi-point scales include increased complexity in completing evaluations, and the ever-present evaluator who “circles down the middle of the scale.”^5^ Studies from core general surgery clerkships suggest that faculty evaluations of clinical performance limited to three points may be as effective as larger scales in predicting a student’s final grades.^4^ Also, internal medicine evaluations of students have shown that breaking down grades to three content areas are also predictive of student performance in several types of evaluations.^6^

However, the emergency medicine (EM) medical education literature contains few publications about the most effective methods to evaluate learners in the clinical setting and the performance of global rating scales in the emergency department (ED) setting.^7^ Therefore, we conducted this study to evaluate the degree that the evaluations provided redundant (as defined by highly correlated) data between the various components of a 10-point global rating scale. A secondary goal of the study was to see if a reduced global rating scale, based on concatenating scores from highly correlated rating items, would provide a similar evaluation of medical students’ overall performance.

## METHODS

### Study Design

We performed a retrospective evaluation on a pre-existing administrative database containing evaluations of medical student performance during their rotation in EM. Institutional review board approved this study with a waiver of consent.

### Study Setting and Population

During the study period, the medical school curriculum included a mandatory EM clerkship in the fourth year of medical school. Students completed 15 eight-hour shifts in the ED and generally worked with a single faculty member for the entire shift. Faculty and third-year residents completed an evaluation card on each student at the end of each shift. Shifts encompassed day, evening, and overnight time periods and included both the adult and pediatric departments.

**Figure 1:**
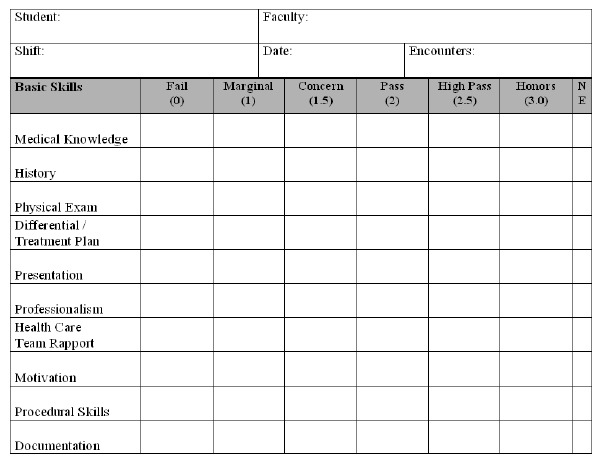
Shift evaluation card. (Reverse side not pictured contained area for written feedback on student’s strengths and areas for improvement).

Our evaluation card contained 10 components used to evaluate the students while in the clinical arena (Figure 1). The card allowed for six grading levels at Fail (0), Marginal (1), Concern (1.5), Pass (2), High Pass (2.5), and Honors (3.0). There was also an option for “Not Evaluated” if the faculty felt they did not have enough information to render an opinion. The reverse side contained space for free-text comments and listing procedures.

During the study period, 25 full-time faculty worked in the ED. All faculty were provided a criterion-based grading scale for the full 10 component evaluation card, based on the six grading levels (Appendix A). We based the criterion-based grading scale on scales used at multiple other institutions. The criterion-based grading scale was reviewed by and with faculty and residents to insure understanding of the scale at faculty meetings and during resident as teacher sessions. Faculty and students were able to access the criterion-based grading scale at any time on the web-based clerkship website. Faculty also received feedback on their grading as compared to all other faculty. The back of the evaluation card provided space for and specifically requested written feedback.

Shift evaluations comprised 65% of the final grade, while a locally developed test provided 25% and adjunct pieces 10% of the final grade. The final grade was determined using criterion-based cutoffs for each of these items. The test (written according to the then current National Board of Medical Examiners question standards) was reviewed for discrimination and reliability by Kuder Richardson (KR)-21, KR-22, and Spearman-Brown statistics. The adjunct pieces included an oral presentation and simulation lab / cadaver lab grades. Demographic data (age, race) were not retained within the database.

### Data Analysis

A correlation matrix using Spearman’s rho was created using the faculty evaluation components to examine interrelationship between responses (Table 1). We observed a natural clustering between certain evaluation components (rho>0.80) in addition to face validity, leading to the establishment of two new variables: clinical skill (composed of a combined average of medical knowledge, physical exam, history, differential diagnosis, and case presentation) and interpersonal skills (combined average of patient rapport, team rapport, and motivation). It should be noted that case presentation also had high correlation with both clinical skills and interpersonal skills; however, it was grouped with the clinical skills variable. Likewise, patient rapport had weaker but still substantial (rho=0.81) correlation with history taking and physical exam; however, the correlations were stronger with the other interpersonal skills, leading us to group patient rapport with team rapport and motivation. Documentation quality and procedural skill did not correlate strongly with other components and were therefore considered in the modeling as separate covariates.

As a sensitivity analysis, to gauge the effect of expected loss of information due to collapsing variables, we constructed separate multiple variable linear regression models using the full model (the weighted average, weighted on number of evaluations, of each of the 10 components) and the reduced model (weighted averages of clinical skill, intrapersonal skill, procedural skill, and documentation quality) in terms of predicting the student’s final grade for the rotation.

We tested each model for normality of the residuals via the Shapiro-Wilk test and heteroscedasticity of the residuals with the Breusch-Pagan test. The adjusted R^2^ was obtained for each model and 95% confidence intervals (CI_95_) were calculated. Given that the sample size was constrained due to the number of students completing the rotation during the study period, we did not conduct formal power analysis. However, given the standard “rule of 10” for regression modeling (10 subjects for every degree of freedom included in the linear regression model), and given that the largest model contained 10 covariates, a minimum of 100 students would be required to avoid potentially overfitting the models. Demographic data (age, race) had not been retained within the administrative database and were therefore not available for inclusion in the modeling process. We calculated statistics using Stata 10.1/SE (College Station, TX). An alpha of <0.05 was held to be statistically significant, and we made adjustments for multiple comparisons in the correlation matrix using the Bonferroni method.

## RESULTS

Over the two academic years the data were collected, 233 students completed the clerkship. The mean number of evaluations per student was 11.7 (CI_95_ (Poisson exact) 11.3–12.1). Both models satisfied the assumptions of normality and homoscedasticity of the residuals. The full model was significantly predictive of the final grade (F_10,222_=5.96, p<0.0001) and had an adjusted R^2^=0.71 (CI_95_=0.65–0.77). The reduced model, using composite variables reflecting clinical skill and interpersonal skill, was likewise predictive of the final grade (F_4,228_=129.64, p<0.0001) and accounted for a similar proportion of the variance in final grade (adjusted R^2^=0.69, CI_95_=0.63–0.76).

## DISCUSSION

Clerkship directors have questioned whether or not evaluators are actually assessing all components of a global rating index of a medical student’s performance or whether they are grouping certain aspects of the evaluation together. By looking at the correlation coefficients of our GRS, there is a strong correlation between the clinical skill components of medical knowledge, physical exam, history, differential diagnosis, and case presentation. The interpersonal skills that had a strong correlation were patient rapport, team rapport, and motivation. The final two components that did not show a strong correlation with each other or the other groupings were procedural skills and quality of documentation. These results are similar to those described by Bandiera et al, although the correlation coefficients in this study signify an even stronger correlation.^7^

Although we can debate the benefit of dividing the clinical skills grouping out into the various components to allow for better feedback to the student on areas of strengths and weaknesses, it appears that the attending or resident’s evaluation does not vary significantly among these components. It may seem to the educator that the evaluation of a student’s physical exam skills is quite different from the student’s medical knowledge assessment, but in reality there is a strong correlation. One argument for this correlation is that the well-performing student does well on all components while the poor performing students stumble on all components equally. It is the authors’ belief that the evaluator develops a global assessment on the student based on overall clinical and interpersonal skills and then links all the grades together in each of these categories.

A well-suited question may be how to provide faculty with the tools to separate these components. Criterion-based evaluation forms define the different components and even the behaviors/qualities associated with each grade level within the scale for each individual component. Despite the fact that we educated faculty and residents on the components and behaviors/qualities appropriate for a given grade level, our data suggest that the evaluation cards submitted after faculty development had high correlation between these components. Whether or not the faculty or residents give directed feedback on these areas during the end-of-shift evaluation discussion is not known.

A secondary outcome of our study shows that combining these components into a four-component evaluation was just as predictive of the final grade. This finding is consistent with research in other clerkships with longer periods (>4 weeks) of evaluator-student interaction which showed a reduced model being as effective in predicting the final grade as a more detailed evaluation form.^4^,^6^ Therefore, based on these data, clerkship directors should be wary of developing lengthy GRSs for faculty and residents to complete on student’s performance. Other means of assessment, such as direct observation, standardized patients, etc. may need to be considered in order to develop a more accurate picture of a student’s ability.

## LIMITATIONS

This study is limited by the fact that we collected data retrospectively. Within the confines of our medical school grading structure, we were unable to directly assess one evaluation scale against the another. We were unable to assess if verbal feedback differentiated among the different components of the evaluation card. Lastly, the GRS used here is not a validated scale.

## CONCLUSION

This study revealed that several components of the evaluation card had a high degree of correlation. This finding calls into question whether a GRS can accurately discriminate between different components. When grouped together into a reduced model containing four components, the evaluation card maintained its predictive level for the final clinical grade. Therefore, when using a GRS for assessment, clerkship directors should evaluate the performance of the GRS in discriminating between components and the feedback provided from the GRS scores.

## Figures and Tables

**Table 1:** Correlation matrix between evaluation category average scores, using Spearman’s rho. All correlations were significant after Bonferroni correction at p<0.0001.

	Medical knowledge	History taking	Physical exam	Differential diagnosis	Presentation	Patient rapport	Team rapport	Motivation	Procedure skills
Medical knowledge	1.00								
History taking	0.91	1.00							
Physical exam	0.89	0.92	1.00						
Differential diagnosis	0.90	0.89	0.89	1.00					
Presentation	0.83	0.87	0.87	0.88	1.00				
Patient rapport	0.77	0.81	0.81	0.77	0.89	1.00			
Team rapport	0.76	0.79	0..79	0.75	0.86	0.93	1.00		
Motivation	0.77	0.79	0.80	0.76	0.83	0.88	0.89	1.00	
Procedural skills	0.66	0.60	0.66	0.60	0.58	0.59	0.59	0.60	1.00
Documentation	0.73	0.73	0.77	0.75	0.75	0.75	0.74	0.73	0.61
